# Plasma proteomics data from hibernating and active Scandinavian brown bears

**DOI:** 10.1016/j.dib.2022.107959

**Published:** 2022-02-16

**Authors:** Anne Mette Frøbert, Simon Gregersen, Malene Brohus, Karen G. Welinder, Jonas Kindberg, Ole Fröbert, Michael T. Overgaard

**Affiliations:** a Department of Chemistry and Bioscience, Faculty of Engineering and Science, Aalborg University, Fredrik Bajers Vej 7H, Aalborg 9220, Denmark; b Department of Wildlife, Fish and Environmental Studies, Swedish University of Agricultural Sciences, Umeå, Sweden; c Norwegian Institute for Nature Research, Trondheim, Norway; d Department of Cardiology, Faculty of Health, Örebro University, Örebro, Sweden; e Department of Clinical Medicine, Faculty of Health, Aarhus University, Aarhus, Denmark; f Department of Clinical Pharmacology, Aarhus University Hospital, Denmark; g Steno Diabetes Center Aarhus, Aarhus University Hospital, Denmark

**Keywords:** *Ursus arctos*, Hibernation, Proteins, Blood, Mass spectrometry, Translational medicine

## Abstract

In this article, we present mass-spectrometry based plasma proteomics data from hibernating and active free-ranging Scandinavian brown bears (*Ursus arctos*).

The brown bear hibernates for half the year. Despite obesity when entering the den and the prolonged period of inactivity, the bear shows no signs of the harmful effects associated with these conditions in humans. Thus, the hibernating bear is a potential translational model for addressing these complications in humans.

We analyzed plasma samples from fourteen 2- to 3-year-old bears (6 males and 8 females) collected both during hibernation and the active state, and for some of the bears during two seasons, resulting in a total of 38 analyzed plasma samples.

In triplicates, the plasma proteins were unfolded and reduced. To increase the chance of detecting low-molecular-weight proteins and peptides, we filtered the samples using a 50 K molecular weight cut-off filter with the aim to deplete larger abundant proteins, including albumin, and thereby increase the depth of the analysis. The proteins in the permeate were then tryptically digested, desalted, and analyzed with liquid chromatography-tandem mass spectrometry (LC-MS/MS). Protein identification and quantification was performed with the MaxQuant software searching against an *Ursus arctos horribilis* protein database.

Here, we provide the raw data, a list with identified proteins in the plasma samples, and the databases applied for protein identification.

Based on the provided data, differentially expressed proteins in hibernation compared to active state can be identified. These proteins may be involved in the bears’ adaptions to hibernation physiology and hold potential as novel therapeutic targets.

## Graphical abstract

**Figure:**
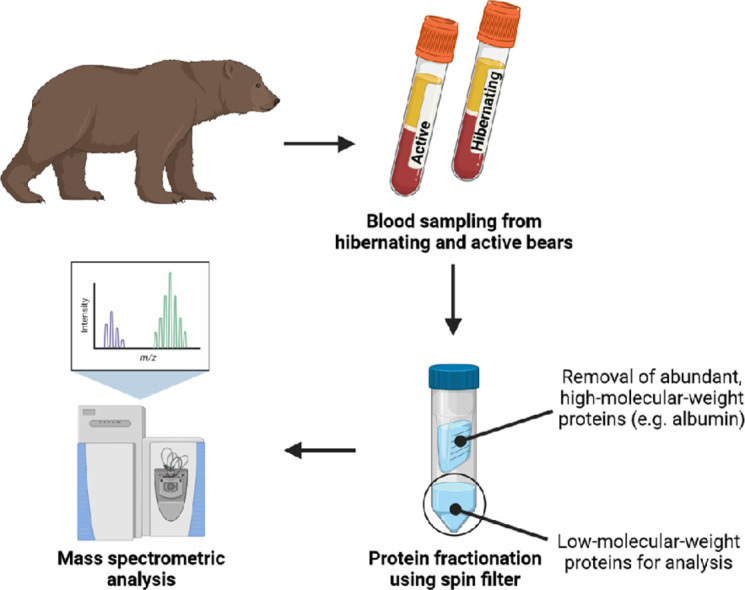

## Specifications Table

**Table:** 

Subject	Biological Sciences
Specific subject area	Mass-spectrometry based plasma proteomics
Type of data	Raw data, tables
How the data were acquired	The data were acquired by LC-ESI-MS/MS by using an EASY-nLC 1200 system coupled to a quadrupole Orbitrap (Q Exactive HF) mass spectrometer equipped with a Nanospray Flex ion source (Thermo Scientific). Protein identification was performed with the MaxQuant software searching against an *Ursus arctos horribilis* protein database from NCBI (taxonomy ID: 116960) and a peptide hormone database generated by manual extraction of the *Ursus arctos horribilis* mature sequences according to the human annotations in UniProt.
Data format	Raw and analyzed
Description of data collection	Plasma was collected from hibernating and active free-ranging Scandinavian brown bears. All plasma samples were analyzed in triplicates. The plasma proteins were unfolded and reduced. To increase the chance of detecting low-molecular-weight proteins and peptides, we filtered samples using a 50 K MWCO filter to deplete larger, abundant proteins, including albumin, and thereby increase the depth of the analysis. The proteins in the permeate were then tryptically digested, desalted, and analyzed with LC-MS/MS.
Data source location	Captures were performed by The Scandinavian Brown Bear Research Project. The bears were sampled in region Dalarna in central Sweden.
Data accessibility	Raw and analyzed mass spectrometric proteomics data and the databases used for protein identification have been deposited to ProteomeXchange: Data identification number: PXD030482 Direct URL to data: http://www.proteomexchange.org/ https://www.ebi.ac.uk/pride/archive/projects/PXD030482

## Value of the Data

• Based on the provided data, differentially expressed proteins in hibernation compared to the active state can be identified. These proteins might be involved in the bears’ adaptions to hibernation physiology.
• These data are valuable for researchers interested in understanding hibernation physiology (e.g. biologists or veterinarians) or in the translational aspects of this to human medicine (e.g. medical researchers or doctors).
• The dataset can be used to identify proteins or pathways, that may be relevant to study in more detail, or to support protein regulations observed by other experimental methods (e.g. transcriptomics, western blotting, or enzyme-linked immunosorbent assay).
• The data can be split according to sex (male vs. female bears) or age (2- vs. 3-year-old bears), to identify sex and age specific protein expression levels. Bears usually reach sexual maturity between the ages of 3 and 5, and therefore the dataset might reveal proteins involved in the transition from cub to adult.
• By appropriate setup of a reanalysis of our raw data, researchers can look for the presence of specific peptides to determine e.g. signal peptide cleavage sites, splice variants, or post translational modifications.

## 1 Data Description

**Fig. 1:**
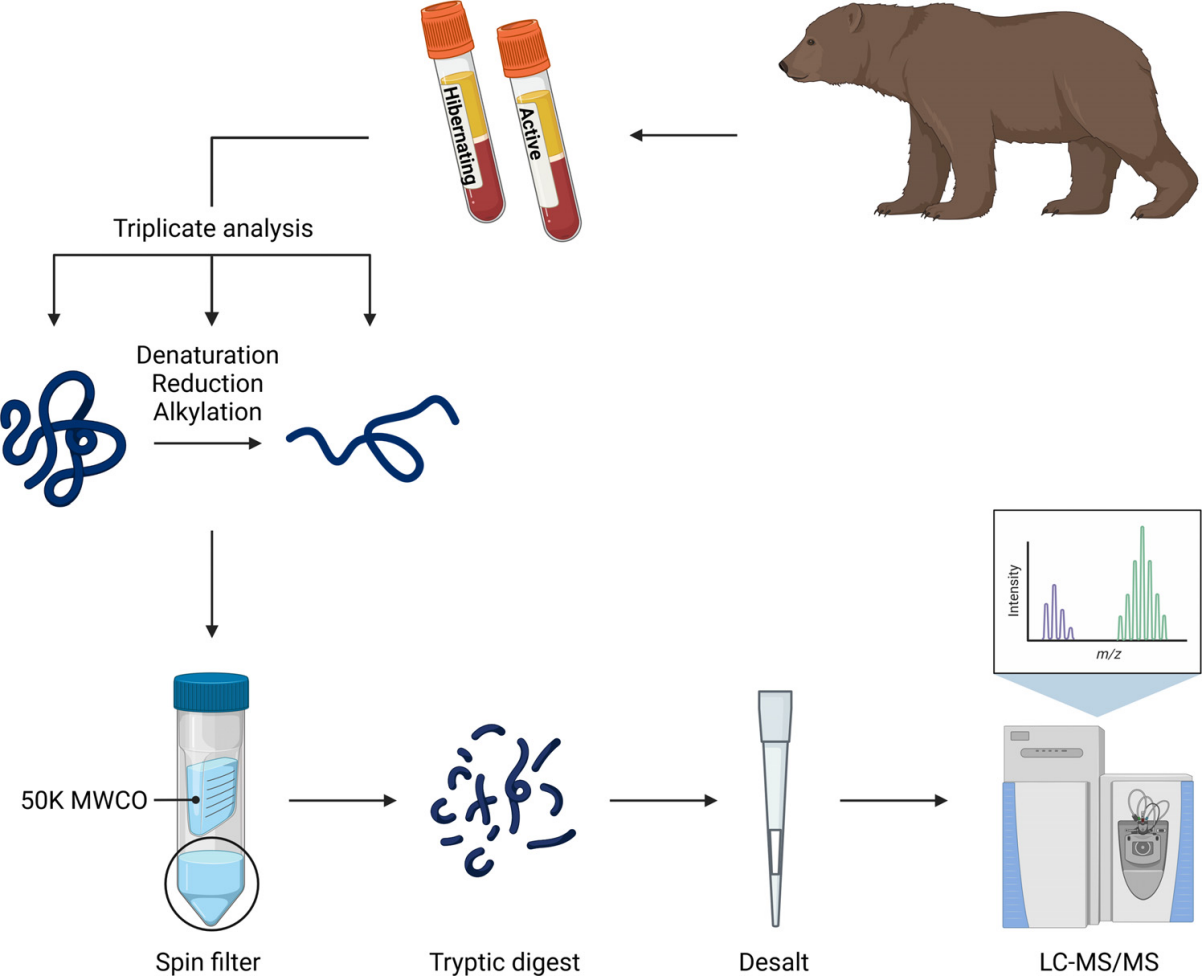
Overview of sample handling. Plasma was collected from hibernating and active Scandinavian brown bears. In triplicates, the plasma proteins were unfolded, reduced, and alkylated. To increase the chance of detecting low-molecular-weight proteins and peptides, we filtered samples using a 50 K MWCO filter. The proteins in the permeate were then tryptically digested, desalted, and analyzed with LC-MS/MS. The figure was created with BioRender.com.

**Fig. 2:**
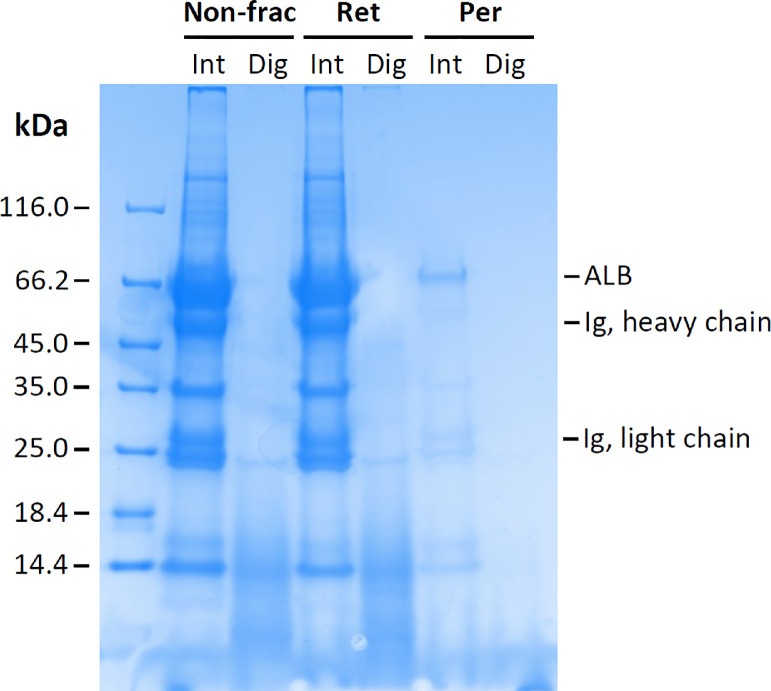
SDS-PAGE analysis (Coomassie Brilliant Blue R‐250 stained) showing the efficiency of sample fractionation using a 50 K MWCO filter to deplete abundant, high-molecular-weight proteins such as albumin (ALB) and immunoglobulins (Ig) which constitute over 75% of the total protein in plasma. The protein content in the samples was analyzed by SDS-PAGE before fractionation (Non-frac) and in the retentate (Ret) and permeate (Per) following fractionation. Additionally, samples were analyzed before (Int) and after tryptic digestion (Dig).

Protein levels in plasma samples from 14 bears collected both during hibernation and the active state, and for some of the bears during two seasons, were analyzed by a mass spectrometry-based proteomics approach. The sample handling is illustrated in Fig. 1. To deplete the sample of larger, abundant proteins and thereby increase the chance to detect low-molecular-weight proteins and peptides, we filtered the samples using a 50 K MWCO filter. The efficiency of the fractionation analyzed by SDS-PAGE is shown in Fig. 2. The Scandinavian brown bear summer and winter plasma proteomes, based on samples from seven bears, have previously been published by Welinder et al.*,* where the retentate following filtration using a 30 K MWCO filter was analyzed [1]. In this study, we aimed to expand the coverage of the bear plasma proteome, focusing on low-molecular-weight protein species. The 38 plasma samples analyzed in this study are listed in Table 1.

The 113 raw files (38 plasma samples analyzed in three technical replicates, except for one replicate, which was removed due to contamination) are available via ProteomeXchange with identifier PXD030482.

Protein identification and quantification from the raw data were performed in MaxQuant [3] searching against an *Ursus arctos horribilis* protein database from NCBI and a peptide hormone database generated by manual extraction of the *Ursus arctos horribilis* mature sequences according to the human annotations in UniProt (available via ProteomeXchange). The MaxQuant output files “proteinGroups.txt” and “peptides.txt” are available via ProteomeXchange. The “proteinGroups.txt” file contains all identified proteins, the number of peptides identified for each protein, sequence coverage, LFQ intensities in each sample, etc., which can be used to investigate protein expression levels. The “peptides.txt” file contains amino acid sequences for all identified peptides, which can be used to investigate e.g. splice variants.

**Table 1:** List of plasma samples analyzed in this study. The internal bear IDs applied in the Scandinavian Brown Bear Research Project are stated in the first column (Wxxxx). The numbers in columns 3–6 indicate the sampling year, e.g., W17 is a winter (February) sample from 2017 and S17 is the summer (June) sample from 2017.

		Plasma samples			
		2 y/o		3 y/o	
Bear ID	Sex	Winter	Summer	Winter	Summer
W1601	Male	W17	S17		
W1610	Male	W17	S17		
W1802	Male	W19	S19		
W1814	Male	W19	S19		
W1910	Male	W20	S20		
W1404	Male			W16	S16
W1909	Female	W20	S20		
W1305	Female	W14	S14	W15	S15
W1509	Female	W16	S16	W17	S17
W1709	Female	W18	S18	W19	S19
W1806	Female	W19	S19	W20	S20
W1813	Female	W19	S19	W20	S20
W1304	Female			W15	S15
W1604	Female			W18	S18

## 2 Experimental Design, Materials, and Methods

### 2.1 Sample collection

Arterial blood was collected in EDTA tubes from free-ranging (wild) Scandinavian Brown Bears in Dalarna, Central Sweden during hibernation (February) and activity (June). Captures were performed by The Scandinavian Brown Bear Research Project (http://bearproject.info/).

Details on the methods of bear capture, anesthesia, and blood sample collection have been published previously [4]. In brief, bears were marked with GPS collars and VHF transmitters to enable localization of the bears. During hibernation, bears were located in their dens and anaesthetized with a mixture of medetomidine, zolazepam, tiletamine, and ketamine. During active state, the same bears were located in their habitat and darted from a helicopter with a mixture of medetomidine, zolazepam, and tiletamine. Anesthesia was antagonized by atipamezole. In winter, the bears were placed back into their dens.

Samples from 14 subadult 2- to 3-year-old (y/o) Scandinavian brown bears (6 males and 8 females) were analyzed in this study (Table 1). The use of subadult animals reduced the risk of interference of pregnancy, sexual activity, and past diseases in the analyzes. The bears included in this analysis were chosen so that samples were collected during two consecutive years, if possible, to enable study of age-related effects on protein levels. Such data were however only available for some of the female bears, since male bears are normally too heavy to handle at age 3. We were only able to obtain samples from one 3 y/o male bear. All other male bears were 2 y/o. Five of the females were sampled for two years both when 2 and 3 y/o (two winters and two summers), while one female was only sampled when 2 y/o, and two female bears only when 3 y/o (Table 1). The samples were collected in the period 2014 to 2020. The blood was kept at 5 °C until centrifugation at 200 g for 15 min, immediately upon returning from the field after 1-2 h. Thereafter, plasma was stored at −80 °C.

### 2.2 Sample preparation

38 EDTA-plasma samples (19 paired sets of winter and summer samples) were prepared for proteomics analysis in triplicates (Fig. 1).

10 µL EDTA-plasma was diluted in 450 µL digestion buffer (1% sodium deoxycholate, 50 mM triethylammonium bicarbonate, pH 8.0). The samples were heated at 99 °C for 10 min and then cooled to < 37 °C. The protein concentrations (mean ± SD) were measured by NanoDrop measurements at A_280_ to 1.36 ± 0.16 µg/µL, corresponding to a total protein amount of 624.0 ± 73.6 µg in the samples.

1 µg tris(2-carboxyethyl)phosphine (TCEP) was added per 25 µg total plasma protein as reducing agent and incubated for 30 min at 37 °C. Then, 1 µg iodoacetamide per 10 µg total plasma protein was added as alkylating agent and incubated for 20 min at 37 °C in the dark.

The sample was transferred to a 0.5 mL 50 K MWCO Amicon™ Ultra centrifugal filter (Millipore) and centrifuged for 30 min and 14 000 g at 4 °C. The protein concentrations in the permeates were measured by NanoDrop at A_280_ to 0.10 ± 0.04 µg/µL, corresponding to a total protein amount of 51.2 ± 22.3 µg in each sample.

1 µg sequencing grade modified trypsin, porcine (Promega) per 50 µg plasma permeate protein was added to the permeate samples and incubated overnight at 37 °C.

Sodium deoxycholate was removed from the trypsinated samples by precipitation by adding formic acid to ∼2.0%, incubating at room temperature for 5 min, centrifuging at 13 000 g for 60 min at 4 °C, and saving the supernatant for desalting.

Plasma protein digests were desalted using in-house prepared StageTips. The protocol was developed with inspiration from that by Rappsilber et al [5]. Briefly, 10 µL pipette tips were plugged with two 3 M Empore™ C18 Extraction disks punched out using a 19 gage blunt ended syringe needle. ∼100 µg Poros™ Oligo R3 resin suspended in acetonitrile was transferred to the column and centrifuged at 4000 g for 1 min. Thereafter, ∼100 µg Poros™ R2 50 resin suspended in acetonitrile was transferred to the column and centrifuged at 4000 g for 1 min. To rinse the columns, 40 µL 100% acetonitrile was loaded to these and the columns centrifuged at 4000 g for 1 min. The columns were equilibrated with 40 µL 5% formic acid and centrifuged at 4000 g for 1 min. The supernatant with the tryptic peptides was loaded to the desalting columns and centrifuged at 4000 g for 1 min. The columns were washed with 40 µL 5% formic acid and centrifuged at 4000 g for 1 min. Finally, the peptides were eluted with 40 µL 80% acetonitrile, 0.1% formic acid into 1.5 mL LoBind tubes (Eppendorf). The samples were dried by vacuum concentration [SPD111V SpeedVac® (Thermo Scientific Savant)] immediately after and stored at −18 °C until analysis ∼2 weeks later.

The peptides were resuspended in 0.1% formic acid, 2% acetonitrile in a volume depending on the protein amount measured in the permeate to obtain a final concentration of 0.1 µg/µL.

### 2.3 LC-MS/MS analysis

The 113 peptide samples (38 plasma samples analyzed in three technical replicates, except for one, which was removed due to contamination) were analyzed by LC-MS/MS as previously described [6]. In brief, LC-ESI-MS/MS analysis was performed using an EASY-nLC 1200 system (Thermo Scientific) coupled to a quadrupole Orbitrap (Q Exactive HF) mass spectrometer (Thermo Scientific) equipped with a Nanospray Flex ion source (Thermo Scientific). 5 µL peptide sample (i.e. 0.5 µg total peptide) was injected at a flow rate of 8 µL/min onto a reverse phase Acclaim™ PepMap™ Nano Trap column [100 µm i.d. × 2 cm, C18, 5 µm, 100 Å (Thermo Scientific)] in solvent A (0.1% formic acid) followed by separation on a reverse phase Acclaim™ PepMap™ RSLC analytical column [C18, 2 µm, 100 Å, 75 µm i.d. × 50 cm (Thermo Scientific)]. Peptides were eluted using a stepwise gradient from 5 to 100% of solvent B (0.1% formic acid in 80% acetonitrile) over 60 min at a constant flow of 300 nL/min. MS was operated in positive ionization mode and data dependent top-20 mode, were the top 20 most intense MS1 parent ions were selected for fragmentation by higher-energy C-trap dissociation (HCD) at 28 eV using an isolation window of 1.2 m/z. A full MS scan in the m/z range 400–1200 was acquired at a resolution of 60,000 at 200 m/z and a maximum ion injection time of 50 ms. MS/MS scans were acquired at a resolution of 15,000 at 200 m/z and a maximum ion injection time of 45 ms. Automatic gain control (AGC) target was set to 10^6^ for MS and 10^5^ for MS/MS. The underfill ratio was set to 3.5%, a dynamic exclusion of 30 s was applied, and Peptide Match and Exclude Isotopes were enabled. Default charge state was set to 2 and charge state exclusion enabled for unassigned precursors or a charge state of 1 or >6.

The raw mass spectrometry data have been deposited to the ProteomeXchange Consortium (http://proteomecentral.proteomexchange.org) via the PRIDE partner repository [2] with the dataset identifier PXD030482.

### 2.4 Protein identification in MaxQuant

The 113 MS raw files were analyzed in MaxQuant software version 1.6.10.43 [3]. Cysteine carbamidomethylation was set as fixed modification and methionine oxidation and acetylation of the protein N-term was set as variable modifications. Digestion was set to Trypsin/P, thus cleavage at the carboxyl side of lysine or arginine, also if a proline follows. Digestion mode was set to semi-specific since the protein database used for identification contains the prepro-protein sequences. Minimum peptide length was 5. The peptide-spectrum match and protein false discovery rate was set to 1%. Match between runs and dependent peptides were selected to increase the number of protein identifications.

The *Ursus arctos horribilis* protein database from NCBI (www.ncbi.nlm.nih.gov) (taxonomy ID: 116960, downloaded on October 29, 2018, 43,159 entries) was applied. Additionally, we applied a peptide hormone database with 256 entries, where we manually extracted the mature hormone sequences from 106 preprohormone sequences based on how the human preprohormones are processed according to UniProt (www.uniprot.org). Some of the preprohormone sequences comprised multiple mature hormones varying in length, splicing, or derived from distinct parts of the prepro sequences. The protein database from NCBI entitled “UrsArc_proteindb_NCBI.fasta” and the peptide hormone database entitled “Mature peptide hormone database from UniProt_Ursus arctos horribilis_22–06–2021.fasta” are available via ProteomeXchange.

The following MaxQuant output files has been deposited to ProteomeXchange:

• proteinGroups.txt.
• peptides.txt.

## Ethics Statements

All animal handling and sampling were approved by the Swedish Ethical Committee on Animal Research and the Swedish Environmental Protection Agency (application numbers C268/12, C 3/2016, and 5.8.18–03,376/2020).

## Funding

This work was supported The Lundbeck Foundation [Grant Nos. R126–2012–12,408 and R286–2018–367].

## CRediT authorship contribution statement

**Anne Mette Frøbert:** Methodology, Investigation, Software, Formal analysis, Writing – original draft, Visualization, Funding acquisition. **Simon Gregersen:** Methodology, Validation, Investigation. **Malene Brohus:** Writing – original draft. **Karen G. Welinder:** Methodology, Writing – review & editing. **Jonas Kindberg:** Resources. **Ole Fröbert:** Resources, Writing – review & editing, Funding acquisition. **Michael T. Overgaard:** Conceptualization, Methodology, Writing – review & editing, Supervision, Funding acquisition.

## Declaration of Competing Interest

The authors declare that they have no known competing financial interests or personal relationships that could have appeared to influence the work reported in this paper.
